# A Cluster-Randomised Trial to Compare Home-Based with Health Facility-Based Antiretroviral Treatment in Uganda: Study Design and Baseline Findings

**DOI:** 10.2174/1874613600701010021

**Published:** 2007-12-13

**Authors:** Barbara Amuron, Alex Coutinho, Heiner Grosskurth, Christine Nabiryo, Josephine Birungi, Geoffrey Namara, Jonathan Levin, Peter G. Smith, Shabbar Jaffar

**Affiliations:** 1 MRC/UVRI Uganda Research Unit on AIDS, c/o Medical Research Council (MRC UK) / Uganda Virus Research Institute (UVRI) P.O. Box 49, Entebbe, Uganda; 2 The AIDS Support Organisation, Old Mulago Complex, PO Box 10443, Kampala, Uganda; 3 Department of Epidemiology and Population Health, London School of Hygiene & Tropical Medicine, Keppel Street, London WC1E 7HT, UK

**Keywords:** Cluster-randomised trial, antiretroviral therapy, effectiveness trial, Africa, Home-based HIV care, baseline characteristics, equity.

## Abstract

The scale-up of antiretroviral therapy is progressing rapidly in Africa but with a limited evidence-base. We report the baseline results from a large pragmatic cluster-randomised trial comparing different strategies of ART delivery. The trial is integrated in normal health service delivery. 1453 subjects were recruited into the study. Significantly more women (71%) than men (29%) were recruited. The WHO HIV clinical stage at presentation did not differ significantly between men and women: 58% and 53% respectively were at WHO stage III or IV (p=0.9). Median CD4 counts (IQR) x 106cells/l were 98 (28, 160) among men and 111 (36, 166) among women. Sixty-four percent of women and 61% men had plasma viral load ≥100,000 copies. Baseline characteristics did not change over time. Considerably fewer men than women presented for treatment. Both men and women presented at an advanced stage with very low median CD4 count and high plasma viral load.

## INTRODUCTION

Antiretroviral therapy (ART) is being scaled-up in Africa but the number of people receiving treatment remains far less than those needing treatment [1]. Major factors inhibiting scale-up include the severe shortage of clinical staff and the restricted access to care due to unavailability or high expense of transportation [2]. There is an urgent need to identify effective models of HIV care that rely less on the expertise of clinical staff, that minimise the demands on existing health services and that do not require patients to travel long distances [3]. What these models should comprise and how they should be evaluated is less clear.

Home-based HIV care, involving drug delivery, monitoring and support of clients in the home by non-clinically qualified staff, could be an important strategy in selected settings [4]. However, it is uncertain as to whether non-clinical personnel can monitor clients on ART adequately and will make referrals for medical care appropriately. Also, regular home visits may be unacceptable because of HIV-related stigma, may be difficult to sustain when clients become healthy and resume mobile lifestyles, and may not be cost-effective.

World Health Organization estimates suggest that an approximately equal number of men and women have been able to access ART thus far [1], but these estimates are derived largely from programmes based in urban centres where most ART roll-out has occurred. Understanding the characteristics of those who access ART in rural and semi-urban areas will be vital in order to assess equity.

We have started a randomised trial in Uganda to compare home-based with the standard facility-based ART delivery. Here we report on the design considerations and baseline results for men and women.

## METHODS

The trial is based at The AIDS Support Organisation (TASO) clinic in Jinja, South-East Uganda and is being conducted in real life health service conditions with TASO staff responsible for all service delivery. TASO is by far the largest provider of ART in the area. Registration at TASO is free for all HIV-infected adults and children. HIV-infected patients were screened for eligibility for ART from August 2004 and recruitment into the TASO ART programme ended in December 2006 when the available funds had been used up. Patients who had been with the organisation the longest were given priority during the first year of the programme and thereafter ART was made available to all on a first come first served basis. Recruitment into the trial began in February 2005 and ended in December 2006 when TASO stopped recruiting further patients. All TASO subjects over 18 years old in whom ART was initiated were invited to join the trial except those living well outside the catchment population or on islands which were more than 100 kilometres away and where provision of home-based care is difficult for TASO. No financial or other incentives to join the trial were provided by the research staff and TASO clinical management procedures were identical for trial participants and non-trial participants. Follow-up in the trial is scheduled to end in 2008.

Patients were assessed and prepared for ART by TASO staff over 3 visits to clinic which were usually spread over 4-8 weeks. Those at WHO late stage III (chronic fever of unknown origin, chronic diarrhoea of unknown origin, oral hairy leukoplakia and pyomyositis), stage IV, or with CD4 count less 200 x10^6^/l were offered ART in accordance with Ministry of Health guidelines. The first line regimen comprised either zidovudine or stavudine, lamivudine, and either nevirapine or efavirenz. Clients were provided with drugs to cover 28 days of treatment and issued with a 7-day pill box (with compartments for morning and evening doses), and trained to transfer the pills from the packets into the pill box. A buffer supply of two days was also provided. Patients were also encouraged strongly to have a medicine companion for support and reminders.

The trial is cluster randomised, comparing two strategies. Treatment was initiated at the TASO Jinja clinic. Patients were then randomised to either home-based or facility-based ART. For those receiving home-based treatment, field officers deliver drugs at home monthly, monitor patients using a checklist and provide adherence support. They refer clients to see a physician at the TASO clinic when judged necessary. Field officers have mobile phones and discuss cases with TASO physicians when they are unclear whether or not to refer. If a client misses a follow-up appointment, the fieldworker returns to the home, usually the next day. If the client is not at home the second time, the fieldworker will leave a message for the client to come to the TASO clinic in Jinja. In addition, clients are asked to come to clinic for routine clinical and counselling reviews at 2 and 6 months after starting therapy and 6-monthly thereafter. In the facility-based arm, clients collect drugs from the pharmacy at the TASO clinic monthly and are seen by a nurse who refers them to a doctor or counsellor if necessary. Clients are also assessed routinely every 3 months by a counsellor and a physician. Clients who miss an appointment are followed-up at home by a fieldworker, usually after 2-3 days, and reminded to attend clinic (provided that the client has given permission for home visits to be conducted).

Clients receiving either mode of care can come to the TASO clinic at anytime they feel unwell. They also have access to a telephone hotline. In exceptional cases, when a client is bed-ridden and unable to travel, home-care is provided by a TASO team including a physician. CD4 count monitoring is done 6-monthly for all clients. Blood is taken for plasma viral load testing, also 6-monthly, but this testing is for research purposes and is done in batches rather than in real-time.

Alongside the provision of ART, TASO offers free voluntary counselling and testing (VCT) to family members of clients on ART. In the facility-based arm, clients are given vouchers for their family members to use at the TASO clinic for VCT. In the home-based arm, VCT is offered in the home at ART initiation or during routine drug delivery visits.

The evaluation was conducted by researchers who were not involved with service delivery. Subjects were interviewed by research staff in private rooms before being seen by TASO clinical staff. Questionnaires were translated into the local language, Luganda, back-translated to English by an independent person unaware of the original English version and cross-checked. All data were double-entered. Analyses were conducted using robust survey analysis procedures that took the clustering due to the study design into account. For categorical variables, the Pearson chi-square statistic for two-way tables was adjusted into an F-statistic with non-integer degrees of freedom, using a second order correction due to Rao and Scott (1981, 1984). For continuous variables, a t-test was done by finding the ratio of the difference of the subgroup mean values to the standard error of this difference, with the standard error being estimated using standard linearization methods for variances in complex surveys. Analyses were done using the statistical package STATA release 9 of Science and Technology and the Institutional Review Boards of the Uganda Virus Research Institute, US Centers for Disease Control and Prevention and London School of Hygiene & Tropical Medicine.

### Trials Endpoints and Sample Size

The trial is designed to test the hypothesis that the effectiveness of home-based care will be approximately equivalent to that of the facility-based care. Following the initiation of therapy, it is expected that the plasma viral load will fall rapidly [5] below detectable limits since this population is almost entirely ART naïve. The primary endpoint is the time for a plasma viral load exceeding 500 copies per ml. Major secondary endpoints include cost-effectiveness and medication adherence.

We assumed that the rate of viral load failure in the facility-based arm would be 10 per 100 person-years at risk. A trial of 40 clusters and 30 subjects per cluster (and a coefficient of variation of 0.2) would provide over 90% power to show that the 95% two-sided confidence interval for the difference between the two arms in the rate of viral load failure lies between ± 3.33 per 100 person-years [6].

### Randomisation

A cluster was defined as a geographical area, usually a sub-county, town, village or a group of villages. Larger areas (e.g. Jinja Town) were divided into more than one cluster, with division made according to major barriers (e.g. a highway). Forty-four geographical areas were defined and randomised in 9 strata according to the estimated number of HIV-infected clients and distance from TASO Jinja.

## RESULTS

4560 adult subjects were screened by TASO. Their median age was 37 (IQR 31, 42) years and 3,368 (74%) were women. Overall, 2636 (54%) subjects were eligible for ART and ART was initiated in 1,889 (41%). The others did not return to clinic. From February 05, 1488 subjects were started on ART. Eleven subjects did not meet the eligibility criteria for the trial and the remainder 1477 subjects were invited to join the trial. Just 24 refused and the other1453 were recruited.

Table **1** shows the characteristics of the trial cohort at baseline. Significantly more women than men were recruited. Women were significantly younger, less educated and less likely to be currently married or living with someone and less likely to be employed. They were also significantly more likely to be renting or living for free and have lower income. There was no significant difference in the time taken to come to clinic or the means of transport used between men and women.

The WHO HIV clinical stage at presentation did not differ significantly between men and women: 58% and 53% respectively were at WHO stage III or IV (p=0.9). More men presented at lower CD4 count category than women but this was not statistically significant (p=0.06, Chi-squared test). Women had significantly lower plasma viral loads than men, with 40% of women having viral loads below 100,000 copies per ml compared to 26% of men (P<0.0001). Twenty-one (1%) subjects had undetectable viral load but reported being antiretroviral naïve.

Women were significantly less likely to disclose to their spouses and partners than men. However, they were more likely to disclose to children and other household members (Table**2**). Women were also significantly less likely to choose spouses as their medicine companions and more likely to choose children than men. The median age (IQR) of children who acted as medicine companions was 14 (11-16) years for men and 13 (11-15) for women (p=0.2).

Understanding of the principles of ART at the time of initiation was generally very high except for side effects in which only an overall 941/1453 (65%) agreed that the ART can make you feel unwell even if the therapy is working. None of the knowledge indicators differed significantly between men and women. Significantly more men than women reported drinking alcohol (47/422[11%] *vs* 80/1031 [8%], p=0.04) and having had sexual intercourse in the last 3 months (183/422 [43%] *vs* 242/1031 [23%], p<0.0001).

The male: female ratio and WHO clinical stage at which men and women presented was unchanged over the 2-year recruitment (Table**3**). There was no association between CD4 at presentation and calendar days since the start of the trial in men (r= 0.025, p=0.6) and women (r= 0.0022, p=0.9). There was a weak association between plasma virus load and days since the start of the trial in both men and women (r=-0.14, p= 0.005 and r=-0.13, p<0.0001 respectively).

## DISCUSSION

The roll-out of ART is progressing rapidly in several African countries and our trial is designed to inform policy, particularly for rural areas where experience with ART is most limited. A number of factors could influence the viability of the trial. There are an increasing number of ART providers (e.g. government, NGO’s, church, private clinics), some with quotas to fill. We may lose participants for follow-up if their health improves on ART and they migrate for work. It is possible that we may have a larger number of withdrawals from the home-based arm because of potentially increased stigma compared with the facility-based arm, although this is likely to occur around the time of randomisation. Those in the home arm may also withdraw in greater numbers as they resume regular employment and find difficulty with being present at home to meet the field officer. The extent of these potential biases is difficult to estimate. We have attempted to minimise risk by selecting an area which has a stable population, where there is one predominant ART provider and where stigma is generally low. A strength of our trial is that it is in partnership between researchers and the health services and is being conducted in close to real life conditions. The results should feed directly into policy and, importantly, trial subjects will receive ART beyond the lifespan of the research as part of the normal health service provision.

This study is being conducted in a poor, largely rural and semi-urban setting and comprising an unselected cohort of subjects coming forward for ART just after ART became available for free in the country. Levels of education, employment, house ownership and income, were low and significantly lower among women than among men. The costs of transport were very high in relation to income, especially for women. Our expectation was that men would access ART from TASO in large numbers, given their higher social status, income and mobility. However, far more women than men accessed the ART service and why men have been reluctant to come forward for treatment is unclear. The proportion eligible for ART in the area was probably similar among men and women since the epidemic in Uganda is old and the prevalence of HIV-infection broadly similar between the two sexes [7, 8]. TASO has been very open to both men and women since it started treating patients with ART and has especially encouraged family members to come forward for screening. We know also that TASO was almost the sole provider of free ART in the district (less than 100 patients were receiving ART from public services during the recruitment period) and so those who did not come forward for ART at TASO are unlikely to have been on treatment. It is plausible that a higher rate of employment among men than women may have made it more difficult for men to come forward for ART although unemployment is generally very high in our setting and employed people are able to access ART from TASO, as is evident from our trial population of whom around 75% were working. Data from rural African settings are scarce but other sites in Uganda have also reported more women than men accessing ART [9]. This requires further research, ideally in an area where demographic surveillance is being conducted and where there is a clear understanding of the demography of the population. Africa may not see the full benefits of ART unless uptake of ART is equitable.

The majority of subjects presented for ART with advanced HIV stage, low CD4 count and high plasma virus load throughout the course of the two-year recruitment period. Mortality and morbidity are very high when CD4 count falls below 200 x 10^6^ cells/l and the complications of treatment are greater when patients start therapy at a more advanced stage. In developed countries, people tend to start antiretroviral therapy at around 250 count x 10^6^ cells/l or higher but there are calls to raise this threshold even higher in an attempt to reduce morbidity and mortality further [10]. It is vital that attempts are made to initiate ART in Africa earlier than at present to ensure that treatment programmes are effective in reducing mortality. Earlier initiation may also reduce infectiousness of HIV-infected subjects and impact on HIV transmission, although this has not been established [11].

There was no evidence that when they presented for ART, men were at more advanced WHO HIV stage or had significantly lower CD4 count than women. Plasma virus load was lower in women but lower levels of virus have been shown in women than in men  [12, 13]. These indicators did not change appreciably over the two years of recruitment for either men or women in our study.

Overall, disclosure rates were very high in this population, and men were more likely to disclose to and select their partners as medicine companions while women were more likely to disclose to and accept children as their medicine companions. There were also high and similar degrees of knowledge about the principles of ART reported by both men and women, suggesting that it is possible to raise awareness and commitment to ART in rural resource-poor settings and in populations which have limited levels of education. Understanding of side effects was less good than the other indicators, but this could be because the terminology “side effects” does not translate well into the local language and may be difficult to study in a quantitative questionnaire format. It will be interesting to see how this level of knowledge translates to adherence and clinical outcomes over time.

The costs of plasma viral load testing are so high that most African countries, including Uganda, do not test routinely and are unlikely to afford these for the foreseeable future, Consequently, we did not provide real time viral load testing. By doing so could influence clinical management and the study results would then be less applicable and of less value to the situation in Uganda and elsewhere in Africa.

In summary, our study shows a significantly higher proportion of women than men accessed ART, and that both men and women present for treatment at a late HIV stage. Research is required to identify the reasons why men are reluctant to access care and to identify ways of reaching and treating patients at an earlier HIV stage. Whether the high degree of knowledge acquired on ART will translate into better adherence and better clinical outcomes remains to be seen

## Figures and Tables

**Table 1:** Characteristics of Men and Women at Baseline

	Men	Women	
Number recruited (%)	422 (29)	1,031 (71)	
Age in years, median (IQR)	40 (34-47)	37 (31-42)	P< 0.0001
Education level, number (%)			
No education	31 (7)	203 (20)	P< 0.0001
Primary	223 (53)	593 (57)	
Secondary	142 (34)	207 (20)	
Tertiary	26 (6)	28 (3)	
Marital status, number (%)			
Currently married/living with someone	279 (66)	237 (23)	P <0.0001
Widowed	53 (13)	530 (51)	
Separated /divorced	86 (20)	245 (24)	
Single/ never married	4 (1)	19 (2)	
Clients’ main occupation, number (%)			
Business/self employed	127 (30)	364 (35)	P< 0.0001
Professional	67 (16)	58 (6)	
Farmer	119 (28)	269 (26)	
Unemployed	102 (24)	294 (29)	
Housewife	0	38 (4)	
Other	7 (2)	8 (1)	
Family owns house, number (%)	231 (55)	462 (45)	P< 0.004
Client’s monthly income			
Overall median (IQR)	30,000 (8,000-80,000)	20,000 (5,000-50,000)	P=0.0005
Time taken to reach clinic, median hours (IQR).	1 (0.5-2)	1 (0.75-2)	P=0. 7
Main form of transport used to come to clinic, number (%)			
Walk	21 (5)	46 (4)	P=0.04
Taxi (bicycle, motorbike, car)	368 (87)	944 (92)	
Own transport (bike, car)	33 (8)	41 (4)	
WHO stage, number (%)			
I	5 (1)	15 (1)	P=0.4
II	173 (41)	469 (45)	
III	208 (49)	464 (45)	
IV	36 (9)	83 (8)	
CD4 count x 10^6^/1 at enrolment			
< 50	142 (34)	301 (29)	P= 0.06
50-99	73 (17)	159 (15)	
100-200	164 (39)	482 (47)	
> 200	43 (10)	89 (9)	
CD4 count x 10^6^/l, median (IQR)	98 (28-160)	111 (36-166)	P= 0.08
Plasma viral load copies/ml, number (%)			
< 1,000	4 (1)	17 (2)	P= 0.0001
1,000-9,999	8 (2)	32(3)	
10,000-99,999	98 (23)	356(35)	
100,000-999,999	284 (67)	548 (53)	
>= 1,000,000	28 (7)	78 (8)	
Plasma viral load copies/ml, median (IQR)	206,102 (97,692-460,612)	147,108 (52,596-343,416)	P<0.0001

**Table 2:** Levels of Disclosure, Support and Knowledge Among Men and Women at Baseline

	Men	Women	
	n. 422	n. 1031	
Disclosed to spouse or partner (among those who had a spouse/partner),number (%)	287/305 (94)	284/347 (82)	P=0.0001
Disclosed to children (among those who have children), number (%)	277/325 (85)	830/910 (91)	P=0.0001
Disclosed to all other household members (among those who have household members), number (%)	317/355 (89)	830/912 (91)	P=0.2
Type of medicine companion chosen			
Spouse/ partner	186 (44)	75 (7)	P< 0.0001
Biological child	64 (15)	409 (40)	
Other relative /household member	154 (37)	497 (48)	
Neighbour/ friend	14 (3)	47 (5)	
Other	4 (1)	3 (0.3)	
Choice of medicine companion if married/have partner			
Spouse/partner	185(66)	73(31)	P< 0.0001
Biological child	32(12)	85 (36)	
Other relative/household member	55(20)	64 (27)	
Neighbour/friend	5(2)	15 (6)	
Other	2(1)	0	
Total	279/404 (69)	237/1031 (23)	
Once a person reaches full health again, he/she can stop taking ART drugs, number disagree (%)	381 (90)	971 (94.2)	P=0.04
ART needs to be taken every day for the rest of your life, even if you feel perfectly healthy, number agree (%)	419 (99)	1,024 (99)	P=1.0
Once ART starts working, a person does not need to wear a condom anymore to prevent spreading HIV, number disagree (%)	407 (96)	965 (94)	P=0.09
The side effects of ART can make you feel ill, even when the drugs are working well, number agree (%)	274(65)	667 (65)	P= 1.0
It is OK to share ART drugs with others in the family who are sick but didn’t get them, number disagree (%)	417(99)	1,003(97)	P= 0.1
It is better to stop taking ART drugs for a few days to let your body rest once in a while, number disagree (%).	401(95)	963 (93)	P=0.5

**Table 3:** Changes in Baseline Clinical Characteristics Over the Duration of Recruitment into the Study

	Men	Women	
Male: Female ratio recruited, number (%)			
Feb 2005-Sep 05	134 (28)	338 (72)	
Oct 2005 May 06	159 (30)	375 (70)	
Jun 06 Dec 06	129 (29)	318 (71)	P=0.9
CD4 count, median (IQR)			
Feb 2005-Sep 05	87 (28-158)	107 (41-161)	
Oct 2005 May 06	97 (25-162)	117 (38-168)	P=0.6
Jun 06 Dec 06	106 (31-162)	112 (34-171)	
Plasma viral load, median (IQR)			
Feb 2005-Sep 05	260,206 (109,224-595,220)	172,620 (61,606-415,428)	
Oct 2005 May 06	182,320 (92,796-343,608)	173,988 (52,716-342,020)	P<0.0001
Jun 06 Dec 06	188,472 (89,272-372,624)	125,288 (45, 792-287,752)	
WHO clinical stage III & IV, number (%)			
Feb 2005-Sep 05	76/134 (57)	186/338 (55)	
Oct 2005 May 06	90/159 (57)	186/375 (50)	P=0.2
Jun 06 Dec 06	78/129 (60)	175/318 (55)	

## ABBREVIATIONS

ART: Antiretroviral therapy
VCT: voluntary counselling and testing
TASO: The AIDS Support Organisation
CDC: US Centers for Disease Control and Prevention
MRC: Medical Research Council
UVRI: Uganda Virus Research Institute
MoH: Ministry of Health
